# Molecular subtypes of clear cell renal carcinoma based on PCD-related long non-coding RNAs expression: insights into the underlying mechanisms and therapeutic strategies

**DOI:** 10.1186/s40001-024-01883-8

**Published:** 2024-05-21

**Authors:** Han Wang, Yang Liu, Aifa Tang, Xiansheng Zhang

**Affiliations:** 1 Department of Urology, The First Affiliated Hospital of Anhui Medical University, Hefei, China; 2 Department of Urology, The First Affiliated Hospital of Shenzhen University, Second People’s Hospital, ShenzhenShenzhen, China; 3 Department of Oncology, Yantian District People’s Hospital, Shenzhen, China; 4 Science and Educational Center of Shenzhen Luohu People’s Hospital, The Third Affiliated Hospital of Shenzhen University, Shenzhen, Guangdong China

**Keywords:** Programmed cell death, Long noncoding RNA, Immune infiltration, Therapeutic response, Clear cell renal carcinoma

## Abstract

**Background:**

PCD-related long non-coding RNAs (PRLs) are rarely investigated in relation to clear cell renal carcinoma (ccRCC). As part of this study, we evaluated the immunological potential of PRL signatures as a biomarker for ccRCC prognosis and immunological function.

**Materials and methods:**

Data were downloaded from the International Cancer Genome Consortium (ICGC) and The Cancer Genome Atlas (TCGA) databases. A Pearson correlation analysis was conducted on the 27 PCD-associated genes to determine whether lncRNAs were significantly associated with PCD. Kaplan–Meier analysis, biological function identification, immune infiltration analysis, estimation of efficacy of immunotherapy and targeted drug screening, and exploration of the landscape of mutation status were conducted by analyzing the risk scores.

**Results:**

Seven PRLs, LINC02747, AP001636.3, AC022126.1, LINC02657, LINC02609, LINC02154, and ZNNT1, were used to divide patients with ccRCC into groups with high and low risk. High-risk patients had a worse prognosis than low-risk patients, according to the results, and the PRL signature showed promising predictive ability. More immune cells were clustered in the high-risk group, whereas the immune cell function of the low-risk group was significantly suppressed. The high-risk group was less sensitive to immunotherapy, whereas the low-risk group had positive responses to most drugs.

**Conclusions:**

Collectively, we established and verified a PRL signature that could competently guide the prognostic survival and immunotherapy of ccRCC. In addition, molecular subtypes were determined for ccRCC based on PRL expression, which may help elucidate the underlying molecular mechanism of ccRCC and develop targeted treatments.

**Supplementary Information:**

The online version contains supplementary material available at 10.1186/s40001-024-01883-8.

## Introduction

Renal cell carcinoma (RCC) is a common malignancy of the urinary system, approximately 70% of RCCs are clear cell carcinomas [1]. With the continuous update and development of clinical diagnostic instruments, the rate of early RCC diagnosis is rising steadily. However, approximately 30% of patients are diagnosed with distant metastases [2]. Although surgery is the primary treatment for RCC, it is sub-optimally managed in patients with distant metastases. Moreover, most chemotherapy and radiotherapy treatments for RCC are currently ineffective [3], and while immunotherapy has been a major breakthrough in RCC treatment, individualized therapeutic effectiveness remains inconsistent and unsatisfactory [4]. Therefore, to maximize the therapeutic effects of treatment, patients must be stratified based on heterogeneity. Clear cell renal cell carcinoma (ccRCC) cells are distinguished by their characteristic morphology, featuring cytoplasm with accumulations of lipids and glycogen, forming clear vacuoles [5, 6]. At its core, ccRCC represents a metabolic disorder characterized by the reprogramming of energy metabolism, driven by mutations in key genes involved in metabolic pathways. This metabolic reprogramming encompasses diverse processes including aerobic glycolysis, fatty acid metabolism, and the utilization of amino acids such as tryptophan, glutamine, and arginine [6]. The metabolic rewiring observed in ccRCC facilitates tumor cell survival under conditions of energy deprivation and hypoxia, enabling the synthesis of essential proliferative components such as proteins, DNA, and membranes. Additionally, it aids in evading host immune surveillance and mitigating oxidative stress. Perturbations in the levels of biochemical enzymes, substrates, metabolites, and end products resulting from metabolic reprogramming serve as valuable diagnostic biomarkers. For instance, overexpression of SETD8 in RCC correlates closely with lipid accumulation, advanced tumor grading and staging, and poor prognosis [7–10]. Furthermore, aberrations in hormone secretion and energy metabolism likely play pivotal roles in the complex process of programmed cell death in tumor cells, intricately linking with tumor initiation, progression, metastasis, and therapeutic response [11]. Moreover, the identification of long non-coding RNAs (lncRNAs) has provided insights into pathway elucidation. Acting as regulators, lncRNAs exhibit extensive and nuanced effects on the metabolic pathways and products of ccRCC, exerting influence either directly or indirectly. This integrated regulatory role underscores their significance in advancing research on tumor metabolism, facilitating the discovery of novel tumor biomarkers, and pinpointing potential therapeutic targets for future investigations [5, 7, 10, 11].

Various cellular processes are regulated by programmed cell death (PCD), and disorders in PCD can cause a number of illnesses, such as neurodegeneration, cancer, and autoimmune conditions [12]. The most intensively studied PCD mechanisms currently, including ferroptosis, necroptosis, cuproptosis, apoptosis and pyroptosis, are considerably related to the regulation of the immunosuppressive and clinical outcomes of cancer treatment methods [13].

Long non-coding RNA (lncRNA)s, which were previously considered as genomic noise, were a kind of mRNA-like transcripts longer than 200 nucleotides [14]. Numerous literatures showed that lncRNAs play a critical role in the progression of multiple tumors, including gastric cancer, hepatocellular carcinoma and renal cell carcinoma [14, 15]. The characteristics of PCD-related lncRNAs in ccRCC is currently unknown. In this research, we aimed to utilize various bioinformatic tools and experimental procedures to explore the role of PRLs in ccRCC.

## Materials and methods

### Data collection and collation

**Fig. 1:**
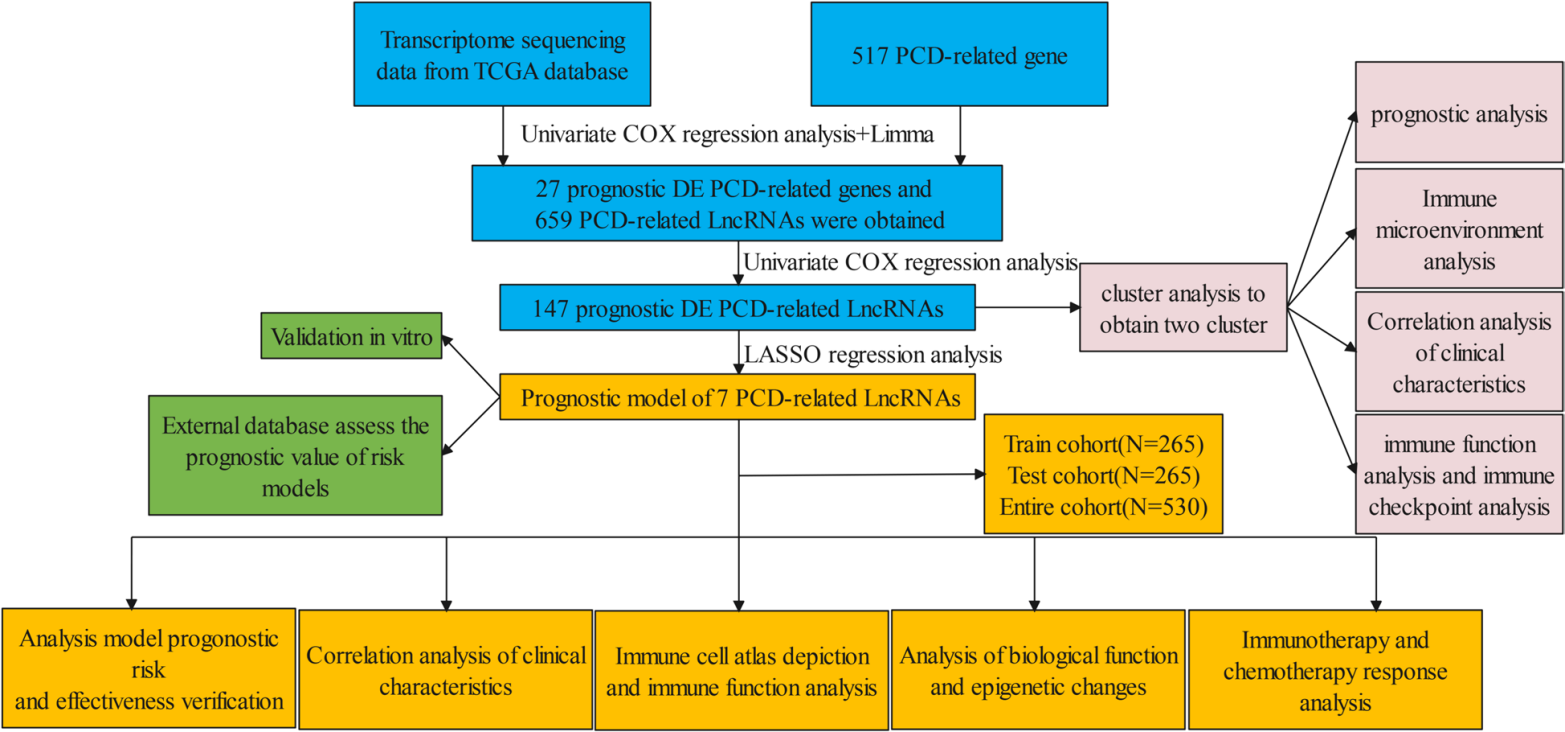
Study workflow

This study’s flow diagram is shown in Fig. 1. The raw data, including RNA-sequencing profiles and specific clinical characteristics of kidney renal clear cell carcinoma (KIRC), were derived from TCGA (https://portal.gdc.cancer.gov) and ICGC databases (https://dcc.icgc.org/releases/current/Projects). To ensure the quality of subsequent analysis, patients with missing clinical data and incomplete prognostic information were removed. Subsequently, 620 KIRC samples derived from the TCGA and ICGC were contained in the study. Transcripts per million were calculated from raw data [16]. Further analysis was conducted on 517 PCD genes, including ferroptosis-related genes [17], apoptosis-related genes [18], cuproptosis-related genes [19], necroptosis-related genes [20], and pyroptosis-related genes [21]. Genes with extremely low expression levels were excluded. Subsequently, 432 PCD genes were included in the downstream analysis.

### Screening and analysis of the prognostic genes

By using the limma package in R, we screened for differentially expressed genes (DEGs) between renal cancer tissues and normal kidney tissues [22]. The screening criteria for the DEGs were performed as follows (Supplementary Table S1): |logFC|> 2.0 and a false-discovery rate (FDR) < 0.05. To identify PCD genes that are related to OS by univariate COX analysis, *p* = 0.01 was used as a cutoff value. To test the correlation between PCD-related DEGs. The interaction network diagram of the proteins was shown by SRTING (https://www.string-db.org/).

### Identification of PCD-related LncRNAs (PRLs)

To identify the PRLs, raw data were downloaded and collated, then the expression data for LncRNAs were obtained using R and Perl program. The lncRNAs that correlated strongly with the 27 PCD genes were identified in ccRCC samples using Pearson correlation analysis (|Cor Pearson|> 0.4 and *p* < 0.001). LncRNAs with differential expression levels were screened out using fold change and *p* values (log_2_ FC > 1, FDR < 0.05).

### Establishment and verification of PRL prognostic signatures

To obtain an effective prognostic prediction model, univariate Cox analysis was conducted to identify PRLs with a considerable association with ccRCC prognosis (*p* < 0.05), following that, 530 samples were randomly split into two subgroups: training (265 patients) and testing (265 patients). Next, the training set data were employed to establish the prognostic model to obtain the prognostic signatures and correlation coefficients, and its validity was also verified simultaneously in both the testing set and the overall dataset. PRLs with mutually high correlations were removed by LASSO regression analysis. Subsequently, using multivariate Cox analysis, independent prognostic factors were identified. To conclude, we created a reliable prognostic prediction model comprising seven PRLs. Calculation of risk scores for patients was carried out according to the following formula:

$$Risk score ={\sum }_{i=1}^{n}coef\left(i\right)*lncRNA\left(i\right)expression.$$

KIRC patients were categorized into high- and low-risk subgroups based on their median risk scores. Kaplan–Meier survival curves were employed to evaluate OS. ROC curves and areas under the curves (AUCs) were used to gauge sensitivity and specificity of the prognostic model. Analyses of univariate and multivariate Cox were conducted to determine whether the risk score and clinical features were independent prognostic factors. Additionally, the prognostic model was validated using ICGC data. Next, a nomogram for 1 year, 3 year, and 5-year OS was generated on basis of gender, age, T, M, tumor stage, risk score, and tumor grade. We evaluated the predicted and practical outcomes using the calibration plot curve analysis.

### Analysis of biological function and immune infiltration level

Limma [22] was selected to screen for differentially expressed genes (DEGs). Then, Gene Ontology (GO) and KEGG analyses were performed using the R packages clusterProfiler, ggplot2, org.Hs.eg.db, enrichplot, and ggpubr [17]. Furthermore, a Gene Set Enrichment Analysis (GSEA) in R was employed to identify the differences in cancer signaling pathways. An immune and stromal score was calculated using the ESTIMATE algorithm for comparison of two subgroups. TIMER [23], XCELL [24], Microenvironment Cell Populations-counter (MCP-counter) [25], CIBERSORT [26, 27], QUANTISEQ [28], and the Estimating the Proportions of Immune and Cancer cells (EPIC) algorithm [29] were applied to evaluate the abundance of immune cells. Finally, two subgroups were assessed using the Wilcoxon test and the GSVA package, respectively, to determine immune checkpoints and immune pathways differences.

### Immunotherapy and targeted drug screening

Data from http://tide.dfci.harvard.edu were downloaded to assess the value of immunotherapy in the different subgroups. A waterfall map depicting the mutational profiles of the two different subgroups was created by Maftools package [30]. Using pRRophetic [20], chemotherapeutic drug sensitivity was assessed for different subgroups of cancer patients.

### Analysis of PRL-defined KIRC subtypes

ConsensusClusterPlus [31] was selected for identification of potential molecular subgroups based on PRL expression. T-SNE and PCA [32] were conducted in R to determine whether the prognostic prediction model could accurately divide KIRC patients into two risk subgroups. Prognostic value, clinicopathological features, immune and risk scores in each cluster were demonstrated by *R*^2^.

### Cell line culture, RNA transcription, and qRT-PCR

Four ccRCC cell lines ACHN, 769-P, 786-O, and CAKI-1, as well as the human kidney cell line 293 T were purchased from the ATCC (Manassas, VA, USA) and cultivated in RPMI-1640 supplemented with streptomycin (25 mg/ml), penicillin (25 U/ml) and 10% fetal bovine serum (FBS). Detailed primer sequences are provided in the Supplementary Material (Supplementary Table S2). RNA transcription and qRT-PCR were operated as described previously [33].

### Statistical analyses

The R version 4.0.5 and the Perl program were applied to process and analyze the data. Single-factor analyses of variance revealed differential expression of PCD-related genes in ccRCC versus normal kidney tissues. Univariate and multivariate Cox proportional hazards regression analysis was performed to estimate the prognostic value. *p* values < 0.05 implied statistical significance.

## Results

### Identification of 27 differentially expressed prognostic PCDs and PRLs in KIRC patients

**Fig. 2:**
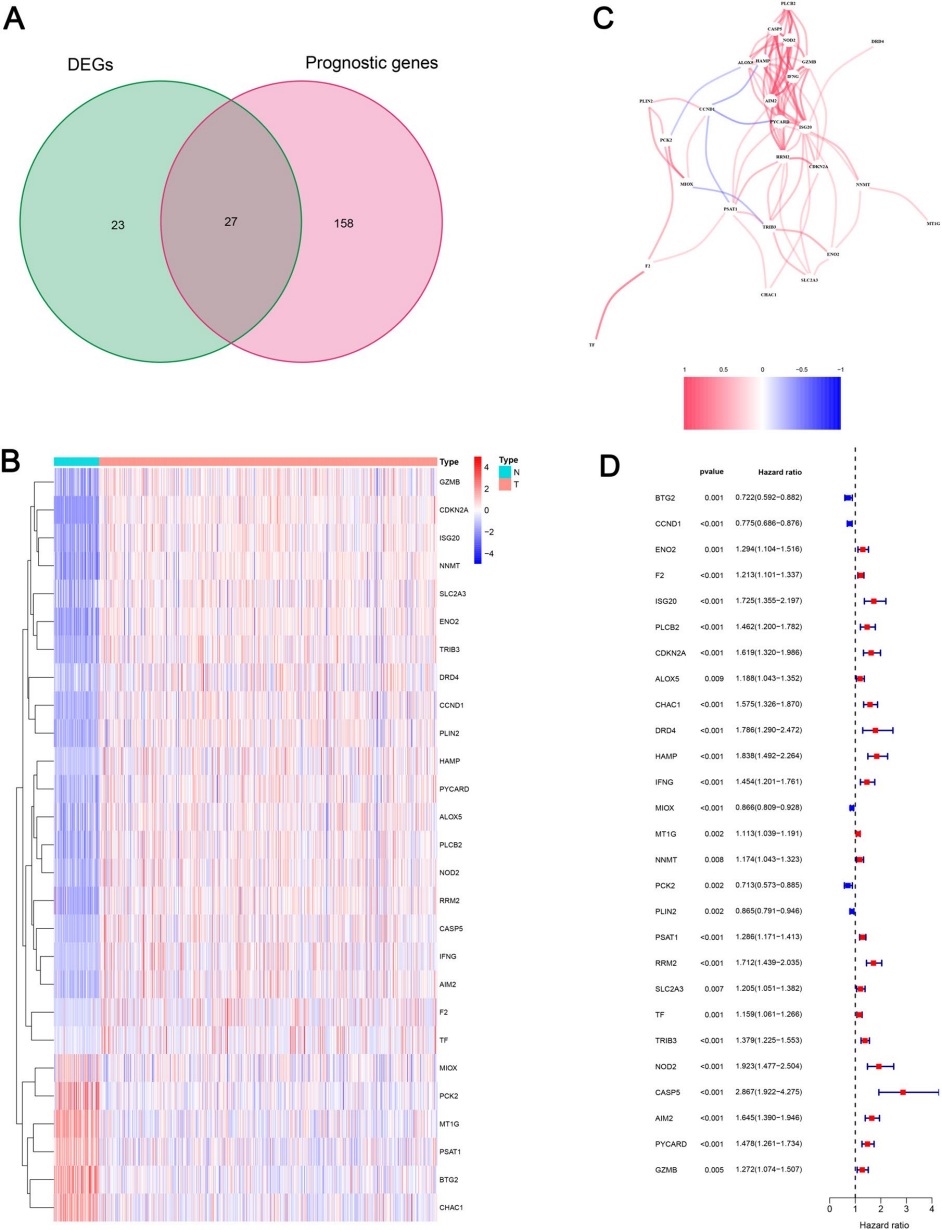
Identification of the prognostic PCD-related DEGs in the KIRC cohort. **A** Venn diagram showing the overlapping genes between PCD-related DEGs and OS-related genes; **B** heatmap showing the candidate genes expression; **C** the correlation network of candidate genes; **D** forest plots showing the candidate genes identified by univariate Cox regression analysis

**Fig. 3:**
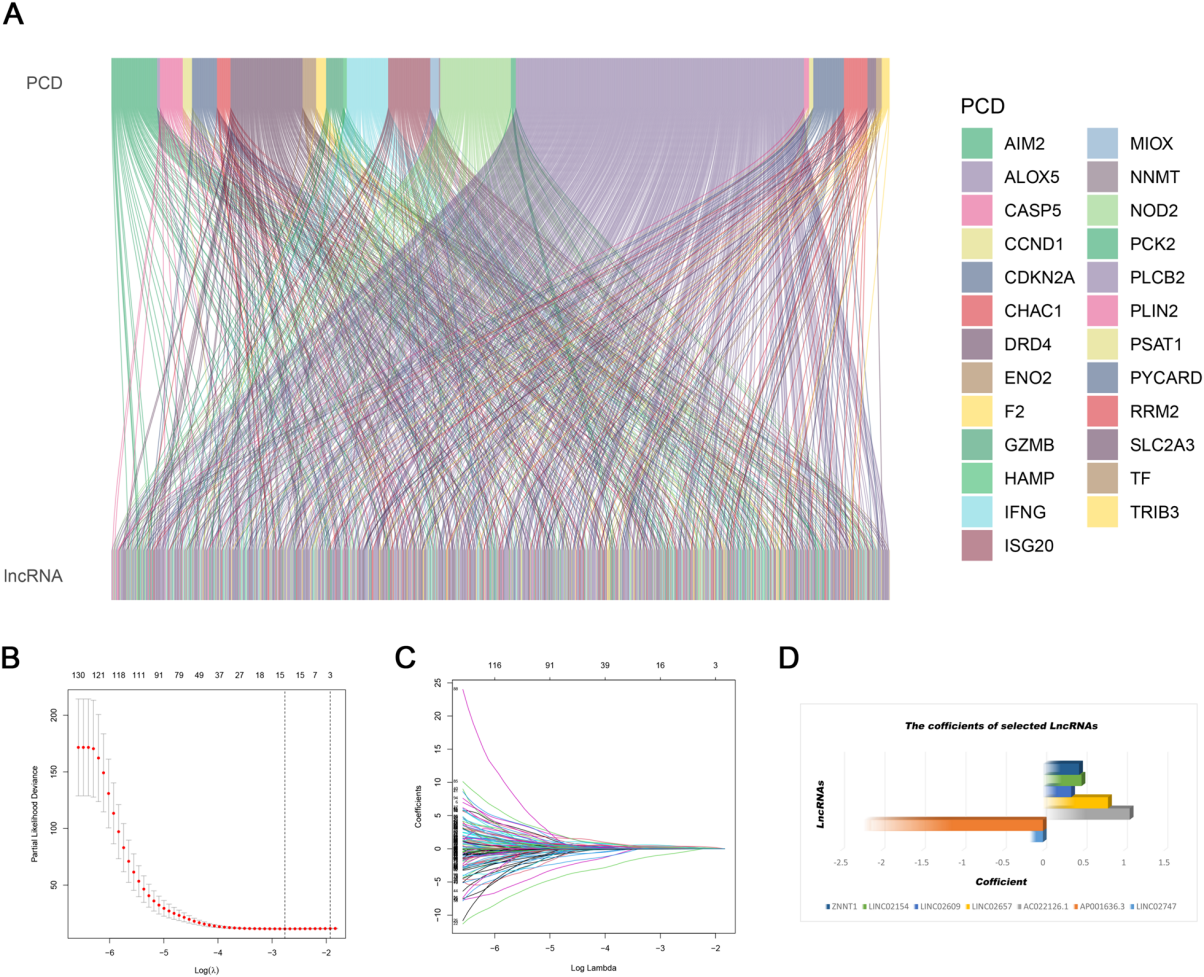
Identification of a prognostic PRL signature in KIRC. **A** Sankey diagram of the relationship between PCD and PRLs; **B** LASSO coefficient distribution of PRLs; **C** variable selection cross-validation in the LASSO analysis; **D** coefficients of the seven chosen PRLs

The screening criteria led to the selection of 50 differentially expressed genes, 27 of which were associated with prognosis (Fig. 2A and D). It is shown in Fig. 2B that 27 intersection genes have different expression levels between KIRC tissues and normal kidney tissues. The interactive information of 27 intersection genes is displayed in Fig. 2C. We then screened 659 lncRNAs that were closely related to the 27 PCD genes (Fig. 3A, Supplementary Table S3), and 491 LncRNAs with considerably differential expression levels were identified (Supplementary Fig. 1, Table S4).

### Developing and validating a PRL prognostic model for KIRC patients

**Fig. 4:**
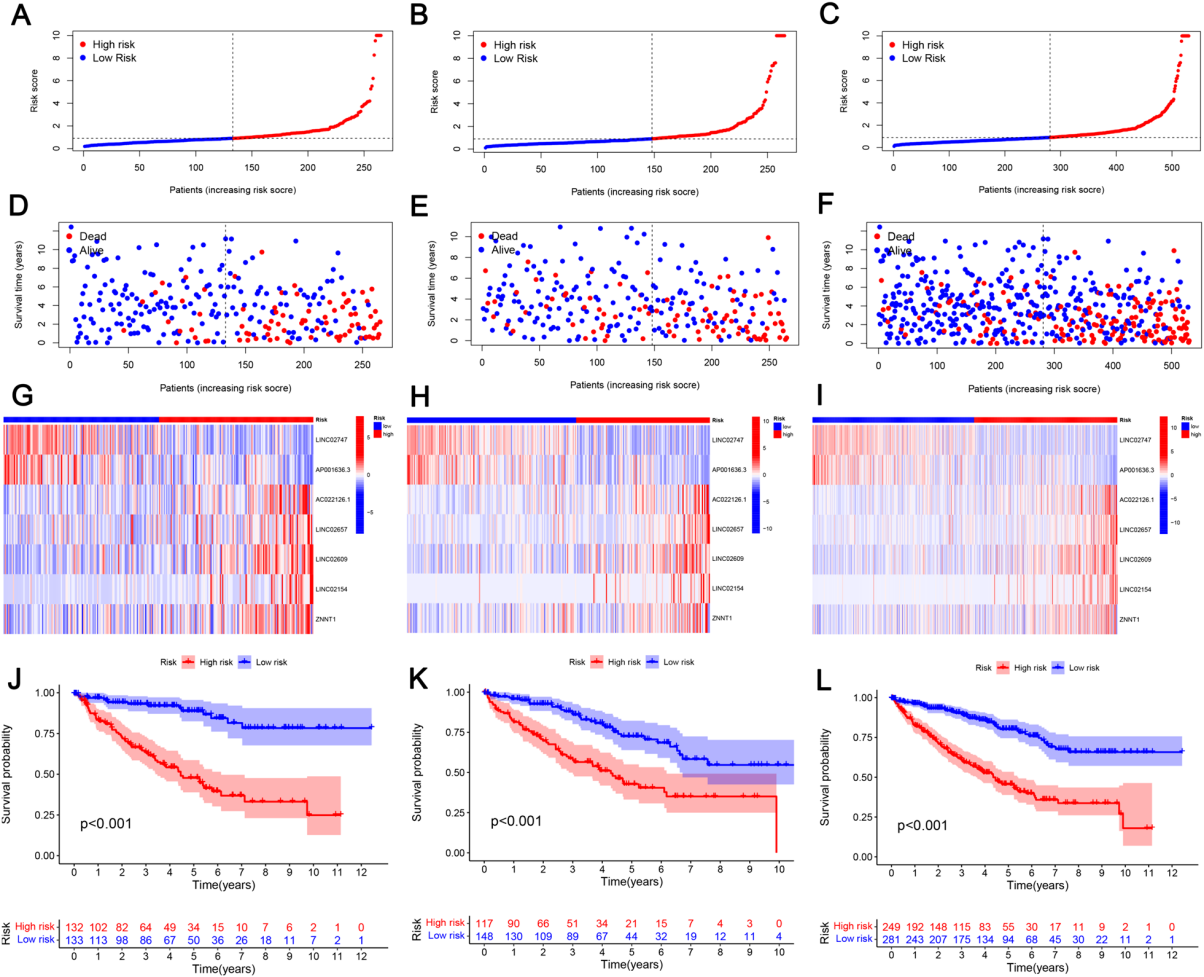
Prognostic analysis of the risk model in different sets. **A**–**C** The overall survival risk score distribution; **D**–**F** the distribution of survival time and survival status; **G**–**I** heatmaps of the expressions of the seven PRLs; **J**–**L** survival outcomes of different sets

**Fig. 5:**
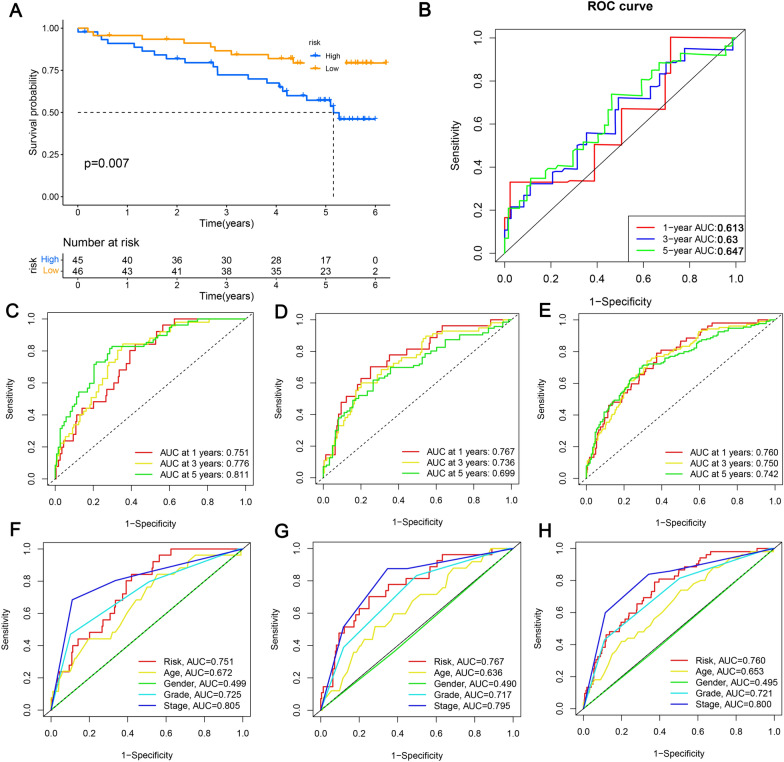
Accuracy assessment of the risk characteristics. **A** Validation of the prognostic signature for KIRC patients in ICGC cohorts; **B** validation of prognostic model effectiveness in ICGC cohorts; **C** evaluation of prognostic model effectiveness in training; **D** testing, **E** overall; **F**–**H** comparison of single prognostic factor and nomogram prognostic efficacy

Univariate Cox proportional hazards regression analyses was employed to determine 147 lncRNAs that were considerably related to the OS (*p* < 0.05), and then multivariate Cox analysis and LASSO analysis were conducted to screen the PRLs. Seven PRLs were used to establish a prognostic risk model: LINC02747, AP001636.3, AC022126.1, LINC02657, long intergenic non-protein coding RNA 2609 (LINC02609), lncRNA associated with SART3 regulation of splicing (LINC02154), and ZNF706 neighboring transcript 1 (ZNNT1). The risk scores of the seven lncRNAs were calculated using their expression levels and regression analysis coefficients (Fig. 3B–D). Risk was calculated as follows: Risk score = LINC02747*(-0.160234181791124) + AP001636.3*(-2.21600604144298) + AC022126.1*(1.07480838933924) + LINC02657*(0.806615089929543) + LINC02609*(0.34964876422504) + LINC02154*(0.478170693602118) + ZNNT1*(0.447721371873328). Low-risk patients in the training set had a notably lower mortality rate than high-risk patients (Fig. 4A and D). Figure 4G illustrates the expression patterns of the seven PRLs in the training set. In the training set, Fig. 4J reveals that the low-risk patients are more likely to survive than high-risk patients. The prognostic risk model was then validated in the testing set (Fig. 4B, E, H, and K), overall set (Fig. 4C, F, I, and L), ICGC set (Fig. 5A), and results from them were highly consistent with those from the training set.

### Analysis of the clinical characteristics of the PRL prognostic risk model

**Fig. 6:**
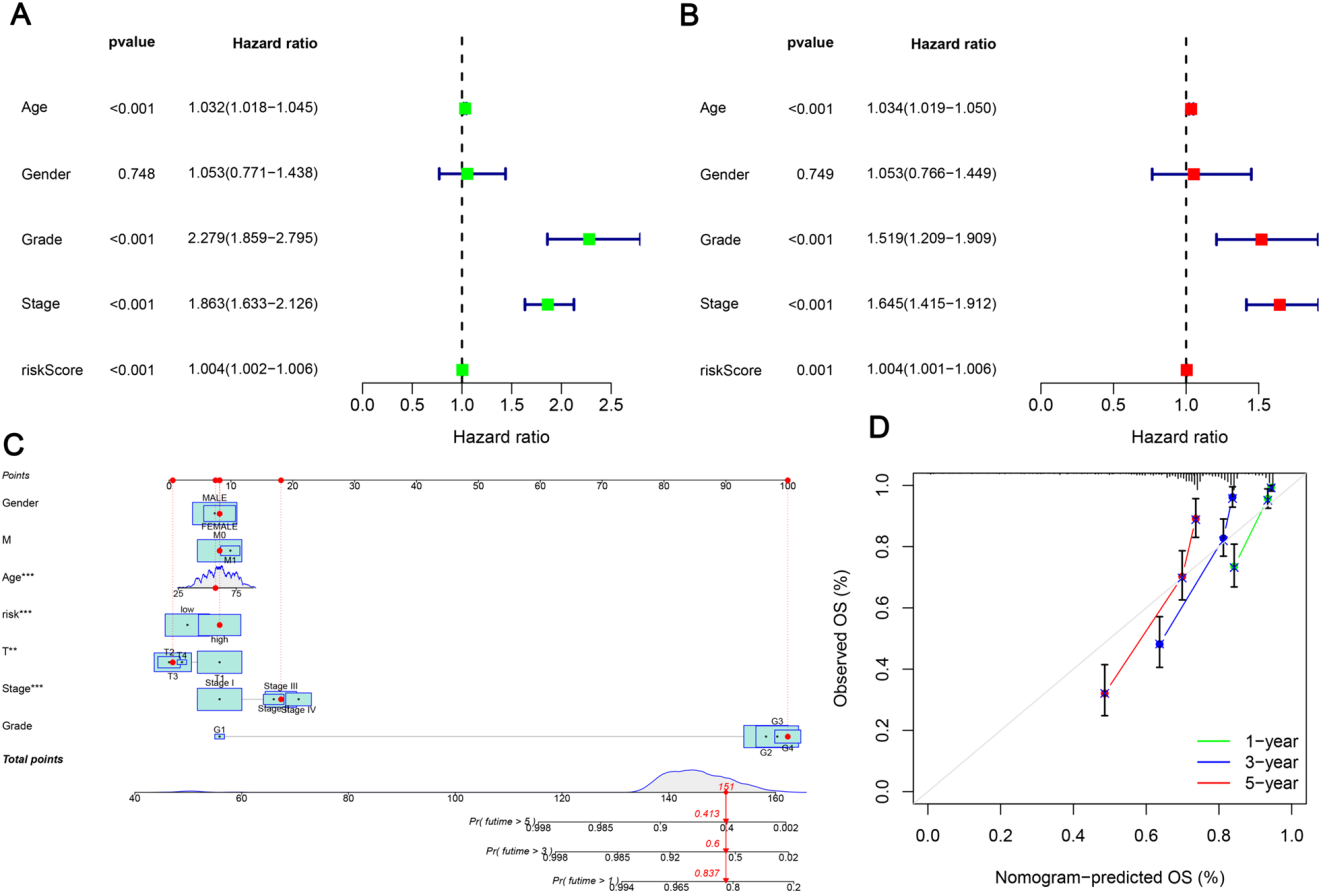
Development of a nomogram by integrating the risk score and clinicopathological features in the KIRC cohort. **A** Univariate Cox analysis; **B** multivariate Cox analysis; **C** nomogram for clinical prognosis assessment (1-year, 3-year, and 5-year); **D** calibration curve to assess nomogram accuracy; *p < 0.05, **p < 0.01, ***p < 0.001, ns, non-significant

In order to evaluate the model’s accuracy, ROC curves and AUC values were calculated. As of the training set, AUC values were 0.750, 0.770, and 0.811 for 1-, 3-, and 5-year OS (Fig. 5C), which indicates that the prognostic risk model we constructed had a promising predictive ability in the training set. Moreover, ICGC data were used to validate the model’s predictive ability (Fig. 5B), the testing dataset (Fig. 5D) and the overall dataset (Fig. 5E). This study also examined whether a prognostic signature could be more accurate at predicting prognosis than common clinicopathological characteristics through multivariate ROC analysis. Based on the training set, AUC value for risk score was 0.751, which was higher than that of age (0.672), gender (0.499), and grade (0.725), but lower than stage (0.805) (Fig. 5F). AUC values for the testing and the overall set are shown in Fig. 5G and H, respectively, which were similar to the training set, indicating that the combination of the prognostic signature and TNM stage may be more valuable for diagnosis. Additionally, as shown in Fig. 6A and B, each of the p values for age, stage, grade, and risk score was less than 0.05, indicating that they could be considered independent variables. In order to facilitate clinical decision-making, a nomogram was constructed based on clinical characteristics and the risk score. Clinicopathological features including age, metastasis, gender, as well as AJCC T stage, grade, stage, and the risk score are all shown in Fig. 6C. In ccRCC patients, the nomogram predicting OS was highly accurate according to the calibration curve.

### Subgroup analysis of prognosis-related clinical features

**Fig. 7:**
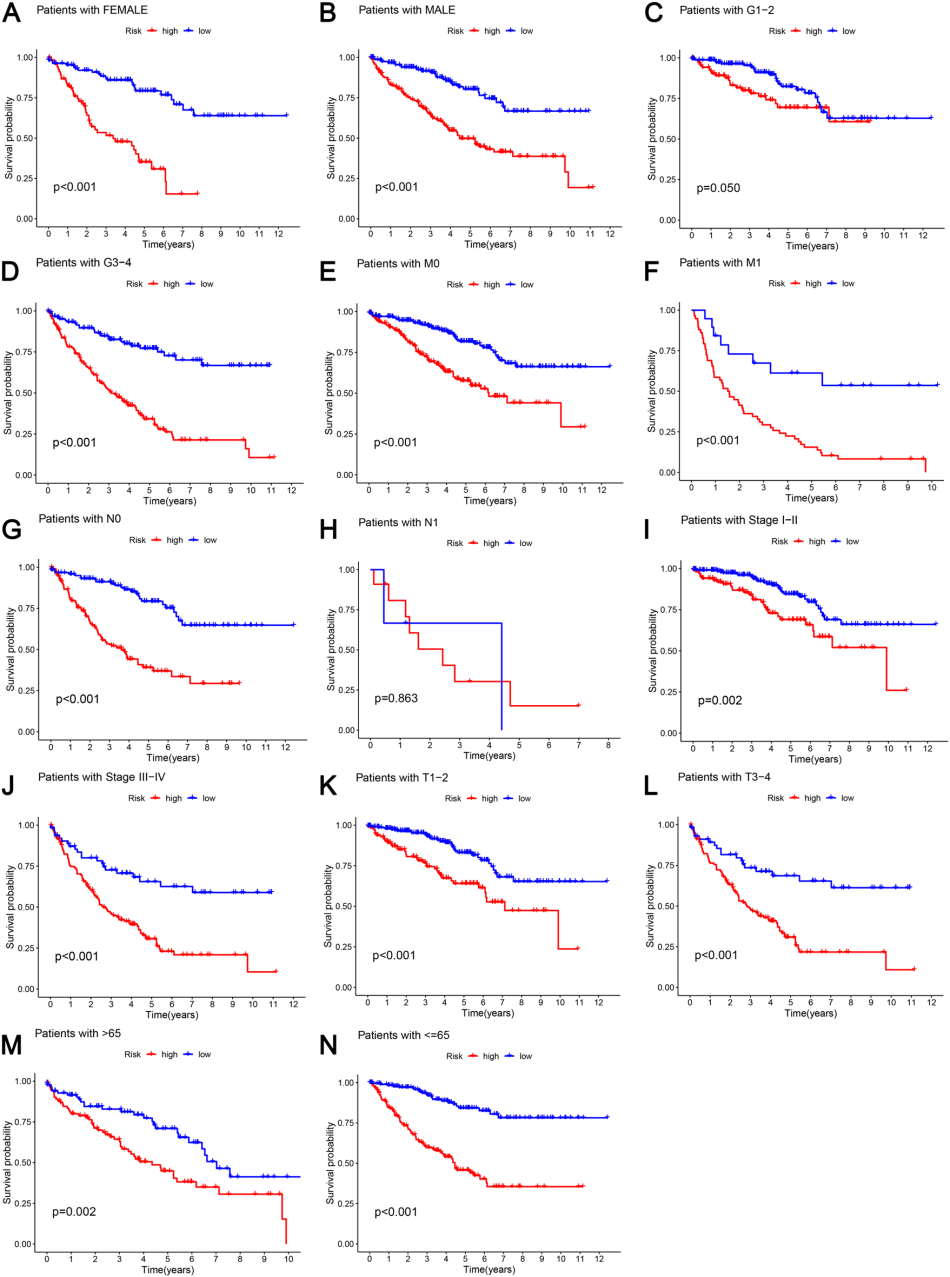
Kaplan–Meier curve analysis for the high- and low-risk groups stratified by clinical features, including **A**, **B** gender; **C**, **D** grade; **E**, **F** **M**; **G**, **H** **N**; **I**, **J** stage; **K**, **L** **T**; **M**, **N**; **D** age

To clarify whether PRLs retain their prognostic ability in different subgroups and to assess their prognostic ability, in order to stratify patients according to their clinical characteristics, we conducted a stratification analysis. In Fig. 7A–N, *p* < 0.05, nearly all low-risk patients had significantly longer OS compared to high-risk patients with varying clinical characteristics, except in Fig. 7H (*p* = 0.863), which might be caused by an insufficient sample size.

### Analysis of biological functions and immune cell infiltration levels

**Fig. 8:**
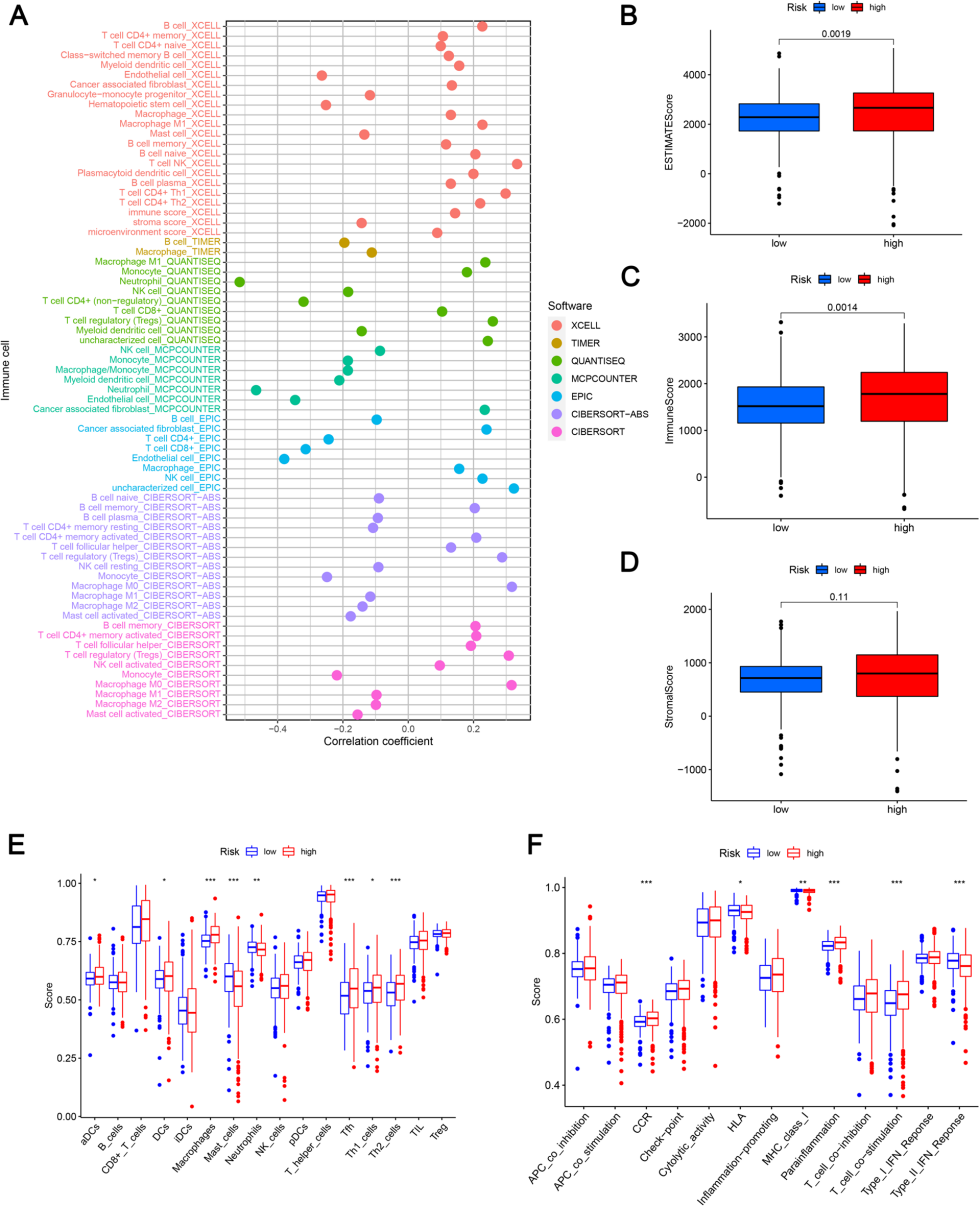
Analysis of immune infiltration in different risk subgroups. **A** Immune cell bubbles of the different groups; **B** ESTIMATE scores of the low- and high-risk subgroups; **C** immune scores of the low- and high-risk subgroups; **D** stromal score of the low- and high-risk subgroups; **E** the ssGSEA scores of immune cells in different risk groups; **F** immune function scores of different risk groups. *p < 0.05, **p < 0.01, ***p < 0.001, ns, non-significant

Aims to identify the potential causes of prognostic differences between two risk subgroups, GSEA enrichment analysis, GO, and KEGG were conducted on the DEGs within the subgroups. As shown in Supplementary Fig. 2A, ERBB, TGF-BETA, JAK-STAT, MTOR, VEGF, MAPK, and WNT signaling pathways were notably enriched in low-risk patients. According to the KEGG analysis, DEGs from the entire population mainly control the interaction between cytokine receptors, complement, and coagulation cascades, and the signaling pathway for IL-17 (Supplementary Fig. 2B). Based on the GO analysis, DEGs had a very high immune pathway enrichment (Supplementary Fig. 2C). Figure 8A depicts the intrinsic link between immune cells infiltration levels and different risk scores. There was a significant positive relationship between the high-risk subgroup and B cells, M0 macrophages, cancer-associated fibroblasts and T cells, as well as a negative relationship between the high-risk subgroup and endothelial and NK cells. Based on Fig. 8B and C, the immune and ESTIMATE scores of the high-risk patients were significantly higher than those of the low-risk patients, however, the stromal score did not show a significant difference (Fig. 8D), indicating that immune molecules were more enriched in high-risk patients. Compared to the low-risk subgroup, high-risk patients exhibited greater immune cell infiltration and immunity (Fig. 8E and F).

### Analysis of immunotherapy and drug sensitivity

**Fig. 9:**
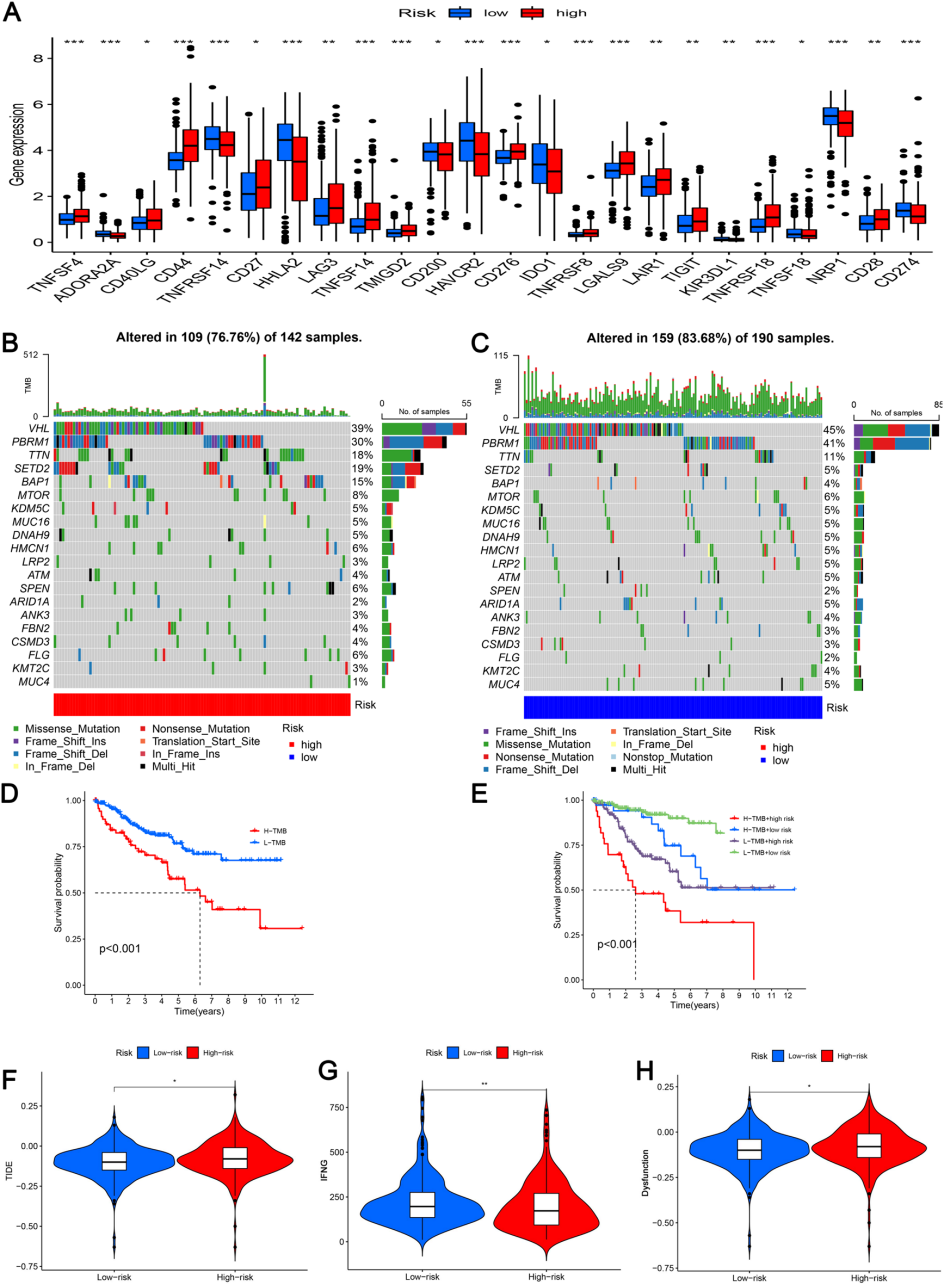
Analysis of immunotherapy. **A** Expression of immune checkpoints in different risk groups; **B** waterfall plot of tumor somatic mutation in the high-risk subgroup; **C** waterfall plot of tumor somatic mutation in the low-risk subgroup, **D** Kaplan–Meier curve analysis of high- and low-TMB groups in TCGA database; **E** Kaplan–Meier curve analysis of TCGA KIRC data stratified by TMB and risk score; **F**–**H** TIDE, IFNG, and dysfunction scores of high- and low-risk subgroups. *p < 0.05, **p < 0.01, ***p < 0.001, ns, non-significant

Given the special status of immune checkpoints in tumor immunotherapy, both risk subgroups were compared regarding their expression levels (Fig. 9A). Compared with the low-risk subgroup, high-risk patients had higher levels of immune checkpoint expression, signifying that immunotherapy might be more effective in high-risk patients. Moreover, as shown in Fig. 9B and C, we investigated whether two different risk subgroups had different somatic mutations and found that the mutation rates of *PBRM1*, *SETD2*, and *BAP1* varied significantly; in particular, the mutation rate of *SETD2* varied by up to 14%, and high-risk patients had a higher frequency of genetic mutations, which might lead to their poor prognoses. To determine whether TMB score correlates with KIRC patient prognosis, a survival analysis was conducted. As shown in Fig. 9D, those with low TMB scores had a better prognosis than those with high scores. As illustrated in Fig. 9E, low PRL risk score groups with low TMB scores had a palpable survival benefit. Meanwhile, the drug sensitivity of the various groups was determined using TIDE analysis, and it seems that immunotherapy may be less effective in the high-risk subgroup as a result of lower scores on TIDE and dysfunction (Fig. 9F and H). Finally, the results of the drug sensitivity analysis showed that patients in the low-risk group were generally sensitive to most drugs, such as sorafenib, paclitaxel, sunitinib, vinblastine, and temsirolimus (Supplementary Figs. 3A–Y).

### Immunotherapy and PRL subgroup generation in KIRC

**Fig. 10:**
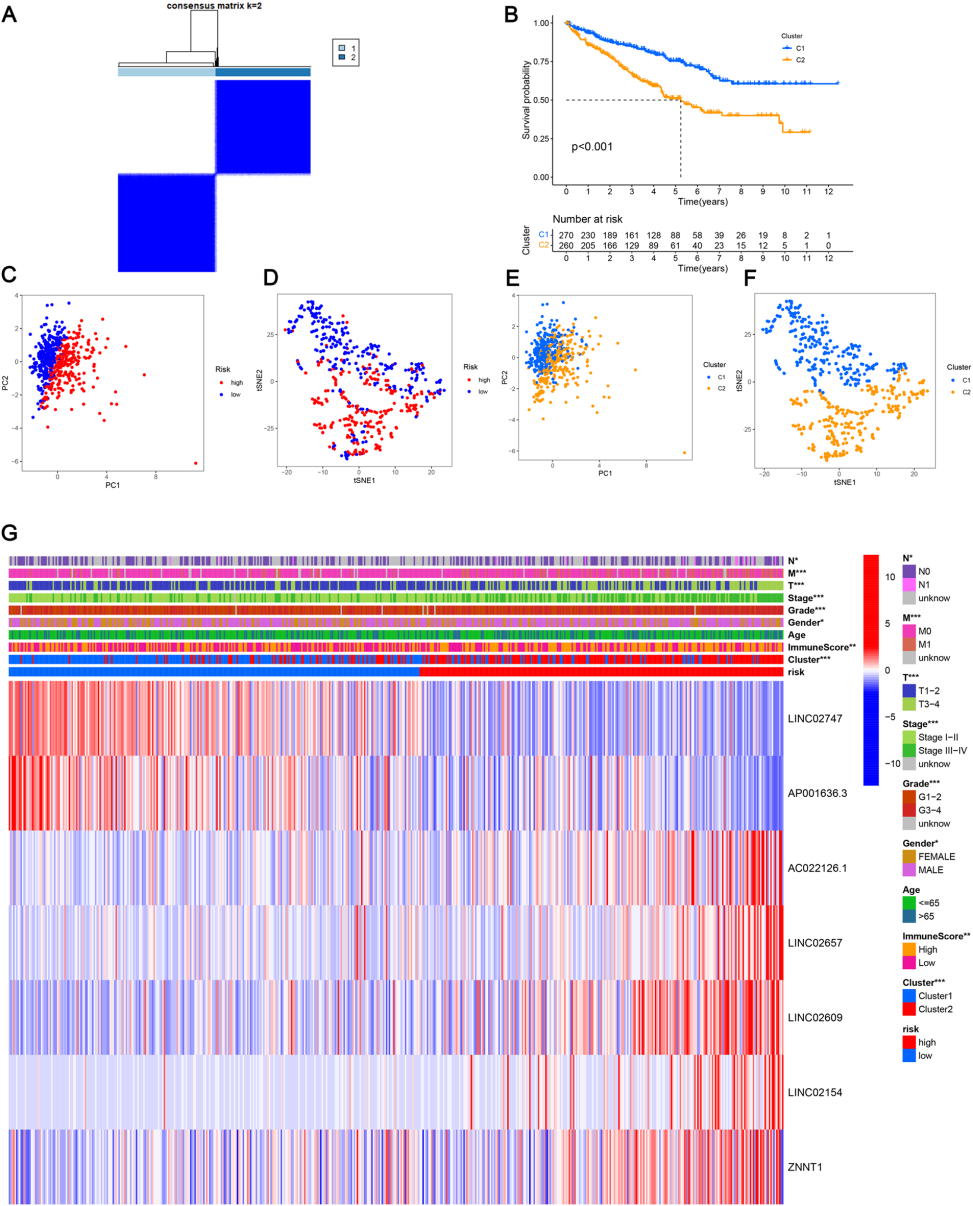
Analysis of KIRC subtypes. **A** KIRC divided into two clusters; **B** Kaplan–Meier curve analysis of OS in clusters; **C** PCA analysis of the two risk groups; **D** t-SNE analysis of the two risk groups; **E** PCA analysis of the two clusters; **F** t-SNE analysis of the two clusters; **G** heatmap of correlations among clinical features, immune scores, and risk scores. *p < 0.05, **p < 0.01, ***p < 0.001, ns, non-significant

**Fig. 11:**
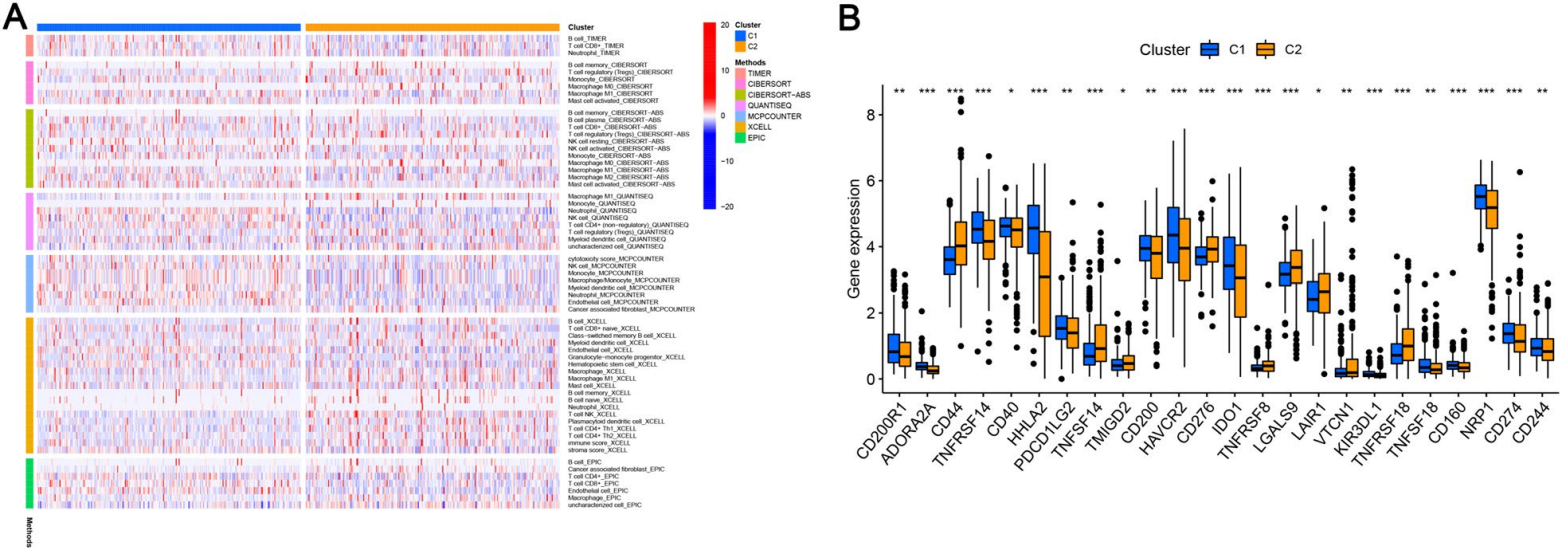
Analysis of immune infiltration in the different clusters. **A** Heatmap of immune cells in the different clusters; **B** expression of immune checkpoints in the different clusters. *p < 0.05, **p < 0.01, ***p < 0.001, ns, non-significant

**Fig. 12:**
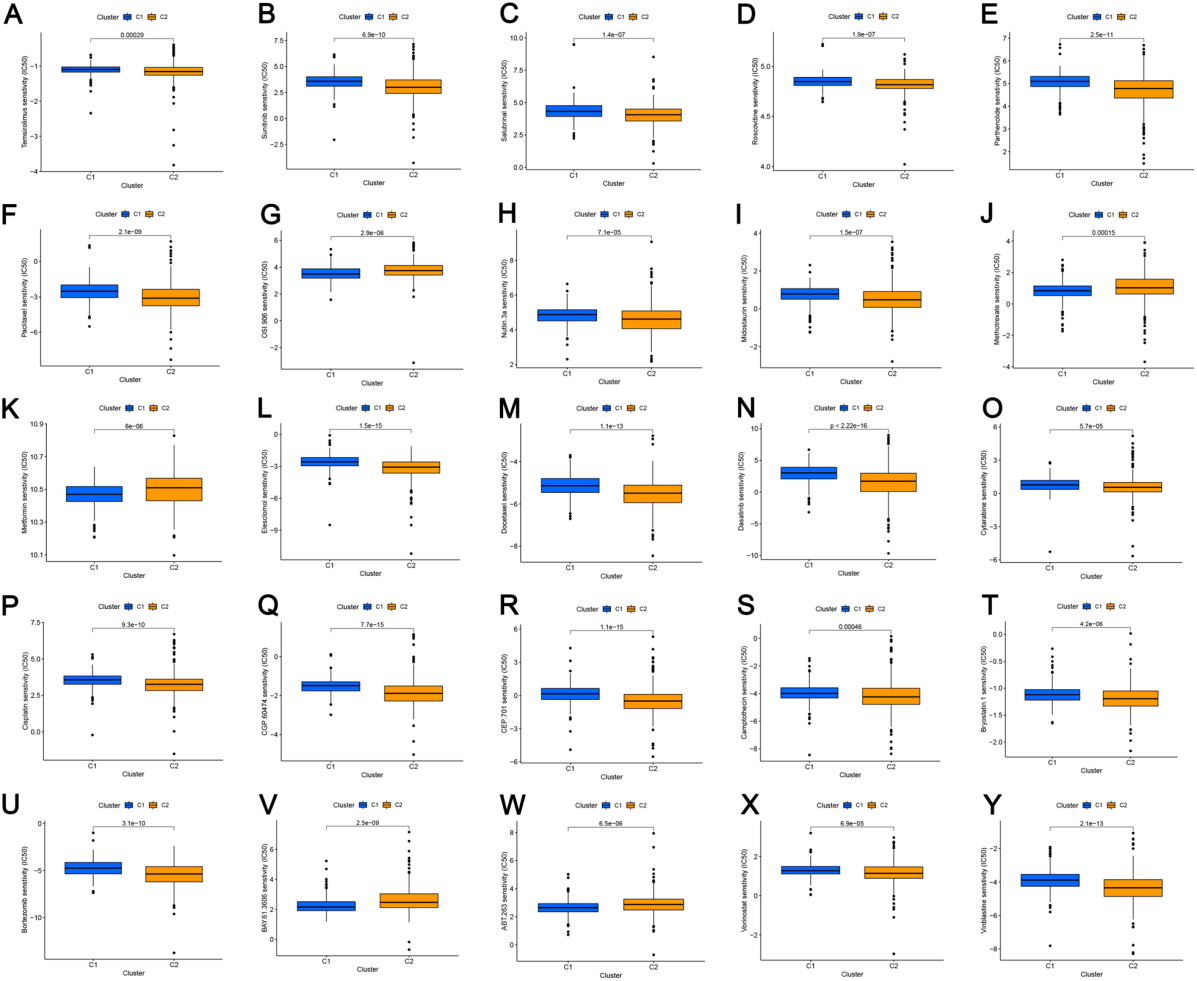
Drug sensitivity analysis in the different clusters (**A**–**Y**)

To explore the relationship between PRL expression levels and KIRC subtypes, KIRC patients were classified using the ConsensusClusterPlus package, and two groups were found to be the optimal clustering method (Fig. 10A). In order to identify clinical differences between the two clusters, as a first step, we compared survival curves of two groups and found significant differences in prognosis (Fig. 10B). Furthermore, using PCA and t-SNE, patients with different risks within clusters were well separated in two directions (Fig. 10C–F). Then, in order to obtain a better understanding of the clinical implications of the seven PRLs in ccRCC, we correlated clinicopathological variables with their expression levels (Fig. 10G). Risk scores varied significantly based on lymph node metastasis, tumor metastasis, cluster stratification, age, gender, AJCC T stage, grade, stage, and immune core. Additionally, tumors in cluster 2 were significantly more likely to be infiltrated by immune cells than tumors in cluster 1 (Fig. 11A). In cluster 1, immune checkpoint expression was higher than in cluster 2, which may have contributed to the lower overall survival of cluster 2 (Fig. 11B). Subsequently, according to our analysis, patients in cluster 1 displayed greater drug sensitivity, such as paclitaxel, vinblastine, sunitinib, sorafenib, and temsirolimus (Fig. 12A–Y). Based on these findings, cluster 1 and low-risk groups should have better outcomes and respond better to immunotherapy.

### Seven PRL expression levels in KIRC cells

**Fig. 13:**
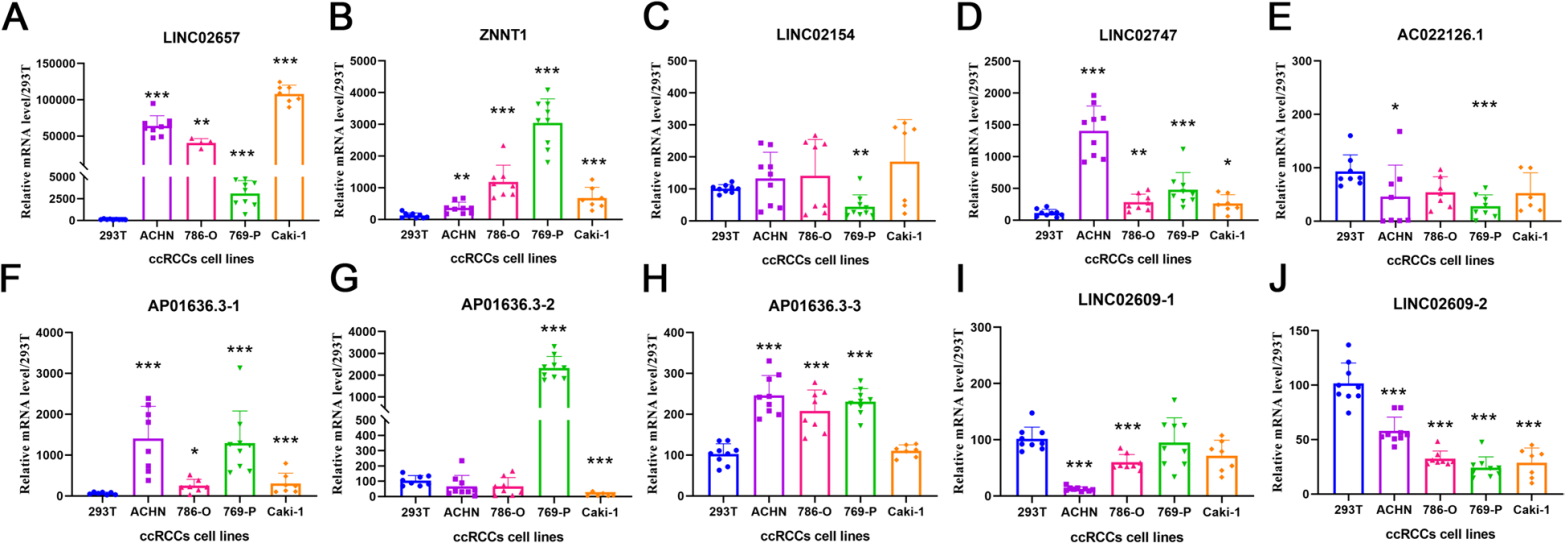
qRT-PCR analysis of the seven PRLs. **A** LINC02657; **B** ZNNT1; **C** LINC02154; **D** LINC02747; **E** AC022126.1; **F**–**H** AP001636.3; **I**, **J** LINC02609. *p < 0.05, **p < 0.01, ***p < 0.001, ns, non-significant

Seven PRLs were determined by qRT-PCR in the 293 T and 4 ccRCC cell lines, 769-P, ACHN, 786-O, and CAKI-1. When compared to 293 T, the expression of LINC02657, ZNNT1, and LINC02747 was considerably higher in the four KIRC cell lines (Fig. 13A, B and D). In contrast, LINC02154 and AC022126.1 were significantly downregulated in KIRC cell lines (Fig. 13C and E). AP001636.3 has three subtypes: AP001636.3–1, AP001636.3–2, and AP001636.3–3, all of which had higher expression in tumor cell lines than in normal kidney cells (Fig. 13F–H). Similarly, LINC02609 has two subtypes, and the expression trends of both subtypes were consistently downregulated in tumor cells (Fig. 13I–J).

## Discussion

Renal cancer, especially ccRCC, usually has a bad prognosis due to its advanced stage with distant metastases [34]. With more treatment options for ccRCC, promising biomarkers are urgently needed to monitor the prognosis of the disease [35]. Clear cell renal cell carcinoma (ccRCC) is fundamentally characterized as a metabolic disorder, marked by a reprogramming of energy metabolism. Transcriptomic, proteomic, and metabolomic analyses of ccRCC tissues delineate the distinct characteristics of metabolic reprogramming. These include the upregulation of aerobic glycolysis (known as the Warburg effect), the pentose phosphate pathway, fatty acid synthesis, glutamine and glutathione metabolism, along with the downregulation of the tricarboxylic acid (TCA) cycle, fatty acid beta-oxidation (FAO), and oxidative phosphorylation. Studies suggest that the downregulation of the TCA cycle, coupled with the upregulation of the pentose phosphate pathway and fatty acid synthesis, may correlate with the aggressiveness of ccRCC tumors and unfavorable patient prognosis [6, 7, 10, 11]. Metabolomic analyses reveal a significant increase in glucose uptake and utilization in ccRCC tumor samples, primarily attributed to compromised mitochondrial bioenergetics and oxidative phosphorylation processes, further facilitated by heightened glucose utilization through the pentose phosphate pathway. Moreover, extensive research indicates the involvement of cell programmed cell death in various tumor metabolic pathways, intricately intertwined with tumor progression [36–39]. According to evidence accumulated over the last decade, tumor progression and metastasis are facilitated by programmed cell death (PCD) [40, 41]. However, most studies have looked at how PCD affects the development of tumors and their treatment, with few studies addressing the prognostic and immunotherapeutic value of PRLs in various cancer. Considering the role lncRNAs play in tumorigenesis and cancer development, a comprehensive investigation of PRLs and their role in renal cancer prognosis and immunotherapy was the goal of this study. This study included various forms of PCD, including ferroptosis, apoptosis, cuproptosis, necroptosis, and pyroptosis.

It was discovered that 27 PCDs and 147 PRLs had significant correlations with OS and would be better predictors of patients with ccRCC. Our prognostic prediction model was constructed using LASSO and univariate Cox analysis and validated with the ICGC dataset. As revealed in survival analyses conducted by TCGA and ICGC, high-risk groups had significantly worse outcomes than low-risk groups. Moreover, a significant difference was found between patients with different risk scores in terms of various clinical characteristics. Furthermore, this model had good predictive value due to its high predictive accuracy and calibration. There has been a great deal of success in predicting the one-, three-, and five-year survival rate by using an independent predictor nomogram (age, AJCC T stage, grade, and prognostic models), which could be utilized to improve individualized therapy and plan short-term follow-up for each patient. The clinical significance of the prognostic signature was also clarified using multiple and univariate Cox analyses, as well as stratification analyses. According to the results, various factors such as age, grade, and risk score were independently associated with a poor prognosis for patients with ccRCC with a high-risk score. Our PRL signature has great value in tumor diagnosis, treatment, and prognosis, which provides a solid theoretical foundation for identifying and treating ccRCC patients effectively. Additionally, in a biological function analysis, various signaling pathways were found to be significantly enriched in the low-risk group, suggesting that a greater influence was exerted by low-risk PRLs compared to high-risk PRLs.

Several biomarkers implicated in our PRL signature have been identified in various cancers. For instance, in ccRCC, LINC02747 acts as an oncogene, upregulating the expression of TFE3 to promote RCC proliferation [42]. It is known that breast cancer promote the activity of SART3 through the action of LINC02651 [43]. Furthermore, in stomach adenocarcinomas and lung cancer, LINC02657 is associated with tumor proliferation and metastasis [44, 45]. LINC02154 accelerates the progression of hepatocellular carcinoma by activating PI3K-AKT [46]. The autophagy-induced ZNNT1 acts as a protective factor against uveal melanoma [47]. There are currently no studies focusing on the diagnosis, prognosis, and therapeutic value of the other lncRNAs, including AP001636.3, LINC02609, and AC022126.1. Preliminary analysis of the seven PRLs in RCC cell lines showed significant upregulation of LINC02657, AP001636.3, LINC02747, and ZNNT1 in the four RCC cell lines, whereas LINC02609, LINC02154, and AC022126.1 were downregulated. Owing to the vital diagnostic and prognostic value of lncRNAs, additional experiments will be required for a better understanding of ccRCC's molecular mechanisms.

Additionally, clear cell renal cell carcinoma (ccRCC) is highly immune-infiltrated and vascularized, representing an aggressive malignancy [48]. Given its resistance to chemotherapy, anti-angiogenic therapy emerges as a primary targeted approach. The tumor microenvironment (TME) significantly impacts tumor biology and therapeutic responses, influencing immune cell function through metabolite dysregulation. Aberrant aerobic glycolysis in tumor cells leads to lactate accumulation and TME acidification, hindering immune cell activity [49, 50]. Understanding immune cell metabolism holds potential for metabolism-targeted therapies, enhancing immunotherapeutic efficacy. Previous studies have demonstrated that T cell regulatory cells, B cell memory cells [51], cancer-associated fibroblasts, and M0 macrophages are related to a poor prognosis of various tumors [52–55]. Our findings are highly concordant with these studies, the poor OS among high-risk patients may be explained by this factor. Furthermore, according to the results of the ssGEA and TME analysis, the immune scores of high-risk participants were higher, as well as the ESTIMATE scores, CCR scores, and T cell co-stimulation scores for the participants. In ccRCC, our characteristics may influence the microenvironment of the tumor immune system in such a way that suppresses the immune response and promotes tumor. Different molecular subtypes have different immune scores, which may result in different prognoses [56, 57]. High-risk patients had significantly higher TIDE and dysfunction scores, indicating that immunotherapy may be less effective, which was consistent with the drug sensitivity analysis. Although our model has good predictive power and can provide effective therapeutic strategies, two clusters of ccRCC patients were identified using an expression level analysis of PRL, to make our model more predictive and provide more precise treatment. In regard to prognosis, the two clusters were significantly different; immune cells were more prevalent in cluster 2, and risk scores varied significantly in lymph node metastasis, tumor metastasis, cluster stratification, age, gender, AJCC T stage, grade, stage, and immune score. Immunotherapy response and prognosis were better in cluster 1 and low-risk groups. Although this study has some strengths. There are also a few limitations to it. First, the model was built and validated by online datasets, necessitating the requirement for prospective research to assess the clinical effect of this model. Second, to elucidate the molecular mechanisms of the seven PRLs in ccRCC, additional experimental evidence is still needed. Thirdly, the interaction between PRLs and the metabolic pathways in renal cancer awaits further experimental validation. However, as a result of our findings, in addition to understanding how PRLs and TMEs interrelate, immunotherapy is also made clearer.

### Supplementary Information

Additional file 1.

Additional file 2.

Additional file 3.

Additional file 4.

## Data Availability

The raw data, including RNA-sequencing profiles and specific clinical characteristics of kidney renal clear cell carcinoma (KIRC), were derived from TCGA (https://portal.gdc.cancer.gov) and ICGC databases (https://dcc.icgc.org/releases/current/Projects). The datasets used and analyzed during the current study available from the corresponding author on reasonable request.
