# Supplementary figures and images for: Molecular subtypes of clear cell renal carcinoma based on PCD-related long non-coding RNAs expression: insights into the underlying mechanisms and therapeutic strategies
Source: Eur J Med Res. 2024 May 21;29:292. doi: 10.1186/s40001-024-01883-8 (PMC11106887; doi:10.1186/s40001-024-01883-8)

A

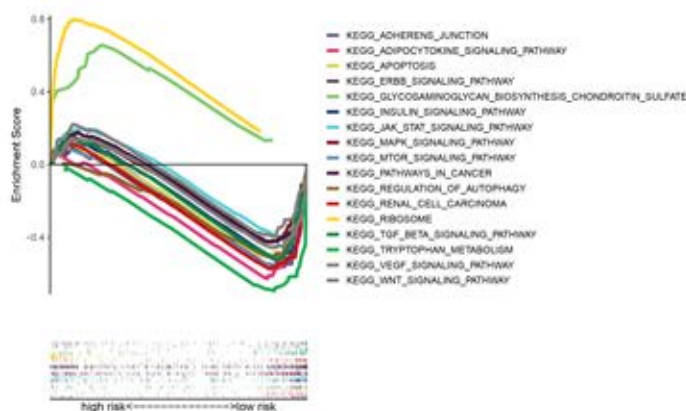

B

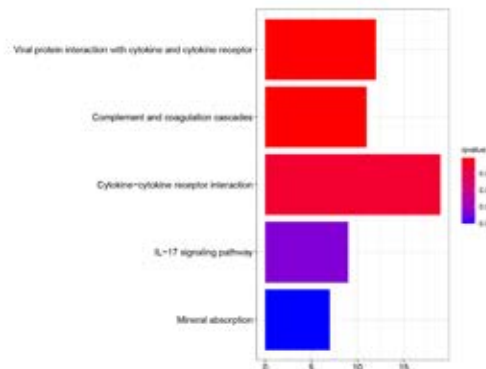

C

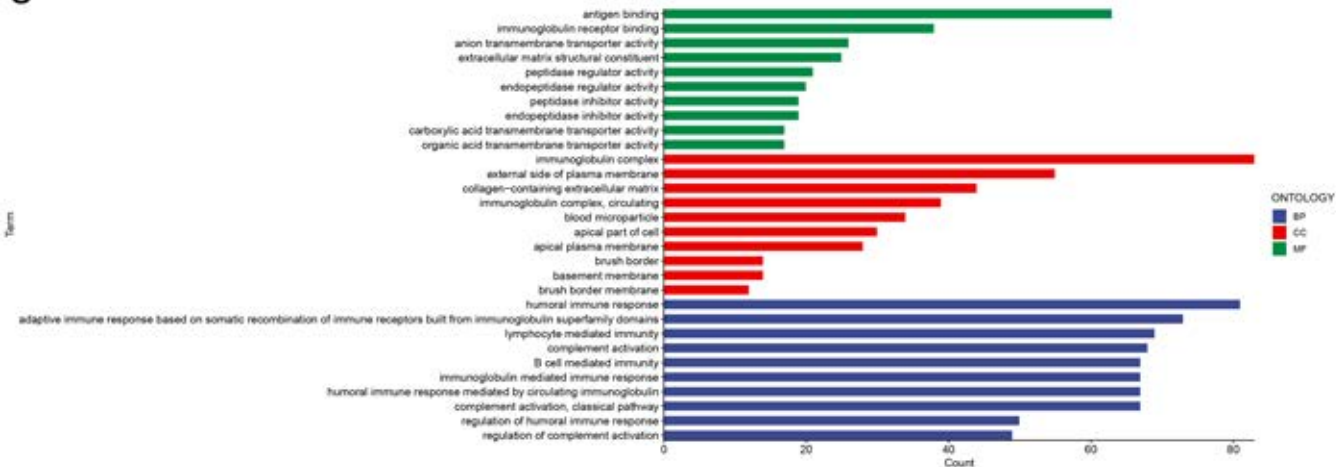

Supplement: Supplementary file 2 — Additional file 2. [file 40001_2024_1883_MOESM2_ESM.pdf]

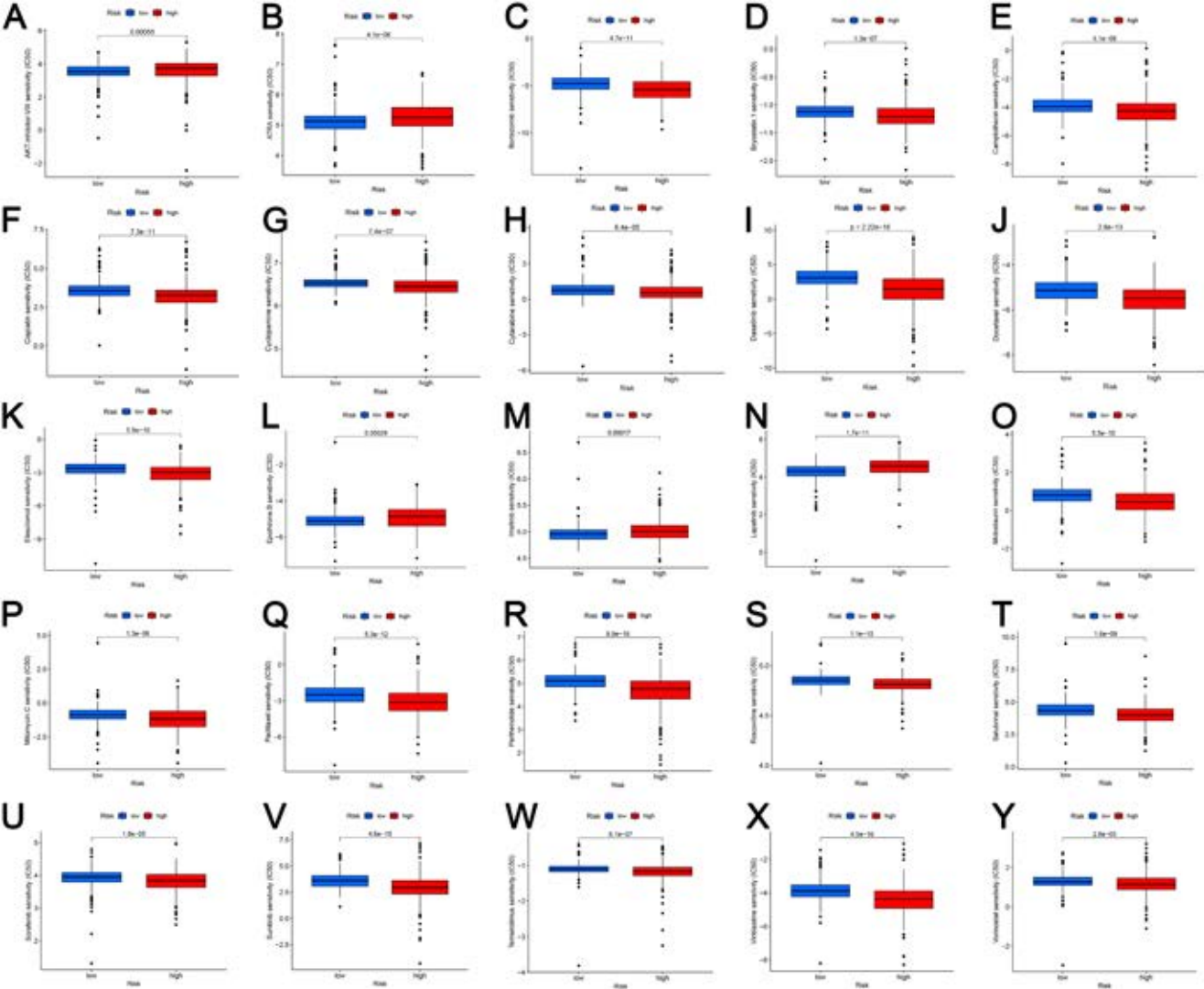

Supplement: Supplementary file 3 — Additional file 3. [file 40001_2024_1883_MOESM3_ESM.pdf]
