# Supplementary material for: Molecular subtypes of clear cell renal carcinoma based on PCD-related long non-coding RNAs expression: insights into the underlying mechanisms and therapeutic strategies
Source: Eur J Med Res. 2024 May 21;29:292. doi: 10.1186/s40001-024-01883-8 (PMC11106887; doi:10.1186/s40001-024-01883-8)
Supplement: Supplementary file 1 — Additional file 1. [file 40001_2024_1883_MOESM1_ESM.pdf]

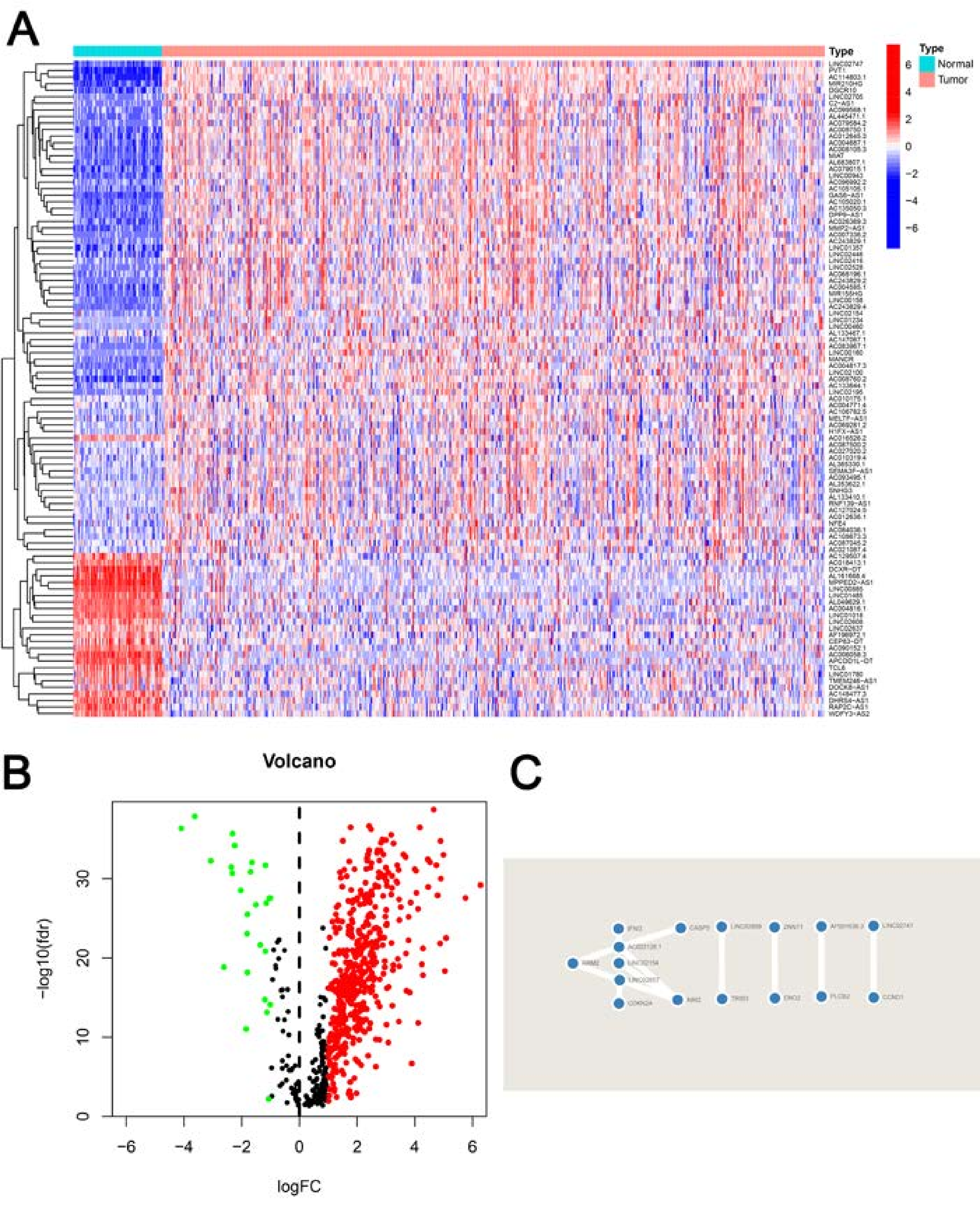

A
Type
Type
Normal
Tumor
6
4
2
0
−2
−4
−6
B
Volcano
-log10(fdr)
logFC
−6
−4
−2
0
2
4
6
0
10
20
30
C
